# Attitudes and practices in the laboratory monitoring of conventional synthetic disease modifying anti-rheumatic drugs by rheumatologists and rheumatology trainees

**DOI:** 10.1186/s41927-022-00290-y

**Published:** 2022-10-17

**Authors:** James J. Tsakas, David F. L. Liew, Cameron L. Adams, Catherine L. Hill, Susanna Proudman, Samuel Whittle, Rachelle Buchbinder, Philip C. Robinson

**Affiliations:** 1 University of Queensland School of Clinical Medicine, Faculty of Medicine, Royal Brisbane and Women’s Hospital, Herston, QLD 4006 Australia; 2 Department of Rheumatology, Austin Health, Heidelberg, VIC Australia; 3 Department of Clinical Pharmacology and Therapeutics, Austin Health, Heidelberg, VIC Australia; 4 Department of Medicine, University of Melbourne, Parkville, VIC Australia; 5 Department of Rheumatology, Royal Prince Alfred Hospital, Camperdown, NSW Australia; 6 Discipline of Medicine, University of Adelaide, Adelaide, SA Australia; 7 Rheumatology Unit, Royal Adelaide Hospital, Adelaide, SA Australia; 8 Rheumatology Unit, The Queen Elizabeth Hospital, Adelaide, SA Australia; 9 Monash-Cabrini Department of Musculoskeletal Health and Clinical Epidemiology, Cabrini Health, Melbourne, Australia; 10 Department of Epidemiology and Preventive Medicine, School of Public Health and Preventive Medicine, Monash University, Melbourne, Australia; 11 Royal Brisbane and Women’s Hospital, Metro North Hospital and Health Service, Herston, QLD Australia

**Keywords:** Methotrexate, Laboratory monitoring, Adverse events, Value-based healthcare

## Abstract

**Objectives:**

There is scant research about laboratory monitoring in people taking conventional synthetic disease-modifying anti-rheumatic drugs (csDMARDs) for rheumatic disease. Our objective was to conduct a scoping study to assess the range of current attitudes and the variation in practice of laboratory monitoring of csDMARDs by rheumatologists and trainees.

**Methods:**

Australian and overseas rheumatologists or trainees were invited through newsletter, Twitter and personal e-mail, to complete an anonymous online survey between 1 February and 22 March 2021. Questions focused on laboratory tests requested by csDMARD prescribed, frequency/pattern of monitoring, influence of additional factors and combination therapy, actions in response to abnormal tests, and attitudes to monitoring frequencies. Results were presented descriptively and analysed using linear and logistic regression.

**Results:**

There were 221 valid responses. Most respondents were from Australia (n = 53, 35%) followed by the US (n = 39, 26%), with a slight preponderance of women (n = 84, 56%), ≥ 11 years in rheumatology practice (n = 83, 56%) and in mostly public practice (n = 79, 53%). Respondents had a wide variation in the frequency and scheduling of tests. In general, respondents reported increasing monitoring frequency if patients had numerous comorbidities or if both methotrexate and leflunomide were being taken concurrently. There was a wide variety of responses to abnormal monitoring results and 27 (40%) considered that in general, monitoring tests are performed too frequently.

**Conclusions:**

The results demonstrated a wide variation in the frequency of testing, factors that should influence this, and what responses to abnormal test results are appropriate, indicates a likely lack of evidence and the need to define the risks, benefits and costs of different csDMARD monitoring regimens.

**Supplementary Information:**

The online version contains supplementary material available at 10.1186/s41927-022-00290-y.

## Key messages

- There was little variation in the types of tests being ordered for DMARD monitoring.
- There was wide variation in the frequency of DMARD monitoring.
- There was also wide variation in the response to abnormal DMARD monitoring tests.

## Introduction

Conventional synthetic disease modifying anti rheumatic drugs (csDMARDs) such as methotrexate, leflunomide and hydroxychloroquine are commonly used drugs in rheumatology. They have both benefits and potential risks including hepatotoxicity, myelosuppression, and nephrotoxicity [1]. Co-morbidities such as fatty liver disease and renal impairment also change the risk profile of individual csDMARDs [2]. Current guidelines for the monitoring of csDMARDs vary, likely due to the lack of high-quality evidence for specific monitoring regimens [3–6]. Some drug monitoring practice recommendations are based on a good knowledge of the relative risk and benefits such as hydroxychloroquine retinal toxicity screening [7, 8]. However, the evidence base supporting csDMARD laboratory monitoring is considerably less robust [1].

The most effective and safe monitoring practice for each drug, indication and co-morbidities has not been established. While some variation is expected, as patient and clinician tolerance of risk will vary in specific clinical situations [3], the optimal balance for patients in general is uncertain. It would be useful to determine what represents excessively frequent monitoring, as such low-value care might not only squander limited healthcare resources, but also cause patient anxiety [9]. Excessively frequent monitoring might also result in unwarranted changes to therapy or trigger unnecessary investigations [10], while insufficiently frequent monitoring may cause a delay in detecting clinically relevant abnormalities.

In the absence of consistent recommendations about how best to monitor for adverse effects, it is likely that practices vary. This may relate to which recommendations rheumatologists follow, if any, as well as how well they adhere to them. Additionally, although a clinician may agree with a guideline this may not translate into adherence to it [11]. In this context, we performed an initial scoping online survey of a convenience sample of rheumatologists and rheumatology trainees. The overall aim was to investigate the range of their csDMARD monitoring practices and attitudes. Specific aims were to (1) Understand what tests were being requested and the range of frequency they were ordered in when they were being used to monitor csDMARDs; (2) Determine how patient co-morbidities and combinations of different drugs influenced monitoring patterns; (3) Determine what actions rheumatologists took in response to abnormal test results and what demographic factors of respondents potentially influenced these responses; and (4) Determine what the attitude of rheumatologists was to both the guideline recommendations regarding how frequent monitoring be performed, as well as to how frequently it was actually being performed in clinical practice, and what demographic factors of respondents potentially influenced these responses.

## Methods

### Design

The survey was developed using SurveyMonkey and made available via a web link from 1st February 2021 to 22nd March 2021. The survey link was distributed via the Australian Rheumatology Association (ARA) newsletter (distributed to ~ 530 Australian rheumatologists and trainees), as well as a convenience sample of rheumatologists identified by the Twitter account of the last author (@philipcrobinson, followers ~ 4500), as well as 25 of his Australian and overseas rheumatologist contacts via personal email. No reminders were sent.

Participants were excluded if they answered ‘No’ to the screening question asking if they are a practicing rheumatologist or rheumatology trainee. There was no exclusion based upon years of practice, sex, primary practice setting or country of practice.

### Survey tool

Items were initially drafted from csDMARD monitoring guidelines published by the American College of Rheumatology (ACR) and the British Society for Rheumatology [4, 12]. The ACR recommendations are to monitor from initiation to 3 months every 2–4 weeks, from 3 to 6 months every 8–12 weeks and from 6 months onwards every 12 weeks [12]. The BSR guidance is similar but monitoring frequency depending on how long specific doses of DMARD are stable for [4]. The main items focused on common monitoring schedules, csDMARDs requiring specific testing, and thresholds for medication review.

We also collected demographic data including sex, country of employment, years in rheumatology practice, clinical setting and the number of half-day outpatient sessions completed on average each week. The survey tool was refined by a panel of five geographically diverse Australian rheumatologists and the final tool included 29 items (See Additional file 1: for a copy of the survey tool).

For each prescribed csDMARD, respondents were asked what blood tests they routinely request, whether they used a fixed or variable monitoring schedule for these requests, and the frequency of monitoring in time periods. If a respondent monitored a patient every two months regardless of treatment duration, this was recorded as ‘one stage’. If they reported that they monitored the patient at monthly intervals for the first three months after initiating a new csDMARD and then reduced the monitoring frequency to every three months from then on this was recorded as a ‘two stage’ monitoring practice etc. A three stage monitoring scheme used three different frequencies of monitoring over three time periods, and a four stage monitoring scheme used four different monitoring frequencies over four time periods. We also explored the impact of polypharmacy and common comorbidities on monitoring practices, and whether trends within the normal range would alter treatment. Respondents were also asked what their laboratory value threshold would be for precipitating a change in medication, their opinion on current guidelines, and how many changes to csDMARD treatment per month they estimate they would make.

### Data analysis

As this was a preliminary exploratory survey of rheumatologist attitudes and practices and a blend of populations were sampled, no sample size calculation was performed and the results are primarily presented descriptively.

When questions generated responses that were proportions, these proportions were compared using the two-proportion Z test with Yates continuity correction implemented. Logistic regression was used to examine the responses to the questions about participants’ attitudes to the frequency of testing stated in guidelines and how often testing was being performed. The two most common responses were used as the dependent variables (‘probably about right’ and ‘too frequent’) and independent variables in the model were country of practice, sex, years of experience, number of times a participant changes a csDMARD dose per month and number of half day outpatient consulting sessions. All analyses were performed in R; linear regression was performed with the lm() function and logistic regression with the glm() function.

### Ethics

Ethical approval was provided by the Royal Brisbane & Women’s Hospital Human Research Ethics Committee (LNR/2020/QRBW/69723). A statement at the start of the survey (see Supplementary data) informed the participant that if they proceeded to complete the survey we would take this as consent to participate. This approach to consenting participants was approved by the ethics committee, therefore we did not seek or obtain written informed consent.

## Results

Of 251 responses, 221 confirmed that they were rheumatologists or rheumatology trainees. Not all respondents completed the full survey. As the demographic items were at the end of the survey, complete demographic details were only available for 150 respondents (67.9%). Based upon the demographic data that were available, the majority of respondents were working in Australia (n = 53, 35%) or the USA (n = 39, 26%), with a wide range of other countries making up the remainder (Table 1). There was a broad representation of primary work environment, experience and gender.

**Table 1:** Demographic details of respondents, N = 150^#^

	N (%)^a^
Country of practice	
Australia	53 (35)
USA	39 (26)
New Zealand	9 (6)
United Kingdom	9 (6)
Ireland	8 (5)
Other (7 or less responses)*	32 (21)
Sex	
Male	84 (56)
Female	66 (44)
Years in rheumatology practice	
< 2 years	7 (5)
2–5 years	32 (21)
6–10 years	28 (19)
11–20 years	40 (27)
> 20 years	43 (29)
Primary work setting	
Private practice	48 (32)
Public practice	79 (53)
Evenly split between public and private	23 (15)

^a^Percentages are all rounded to the nearest whole number (including 0 and 100) and thus some differing raw numbers show the same percent and non-0 raw numbers may show 0%

^#^Demographic data unavailable for whole cohort (N = 221)

*Canada, Italy, Denmark, Latvia, Philippines, Bangladesh, Columbia, Dominican Republic, Ecuador, Finland, France, Germany, Greece, Honduras, India, Mauritius and Mexico

Almost all respondents indicated that regular blood monitoring in an uncomplicated patient on a range of commonly used csDMARDs would include liver function tests (LFTs), full blood count (FBC) and renal function assessment (Table 2).

**Table 2:** Number (percent) who reported requesting full blood count, renal and liver function tests by csDMARD, N = 210^#^

csDMARD	FBC (N, %)*	eGFR (N, %)*	LFT (N, %)*	None (N, %)*	Drug not prescribed (N, %)*
Methotrexate	209 (100%)	199 (95%)	210 (100%)	2 (1%)	0 (0%)
Leflunomide	205 (98%)	186 (89%)	204 (97%)	1 (0%)	5 (2%)
Sulfasalazine	204 (97%)	175 (83%)	201 (96%)	3 (1%)	3 (1%)
Hydroxychloroquine	76 (36%)	68 (32%)	60 (29%)	128 (61%)	0 (0%)
Azathioprine	208 (99%)	187 (89%)	206 (98%)	1 (0%)	2 (1%)
Mycophenolate	202 (96%)	185 (88%)	195 (93%)	2 (1%)	8 (4%)
Tacrolimus and other Calcineurin Inhibitors	135 (64%)	134 (64%)	129 (61%)	3 (1%)	74 (35%)

*FBC* full blood count, *eGFR* estimated glomerular filtration rate, *LFT* liver function tests

^#^Laboratory monitoring data unavailable for whole cohort (N = 221)

^*^Percentages are all rounded to the nearest whole number (including 0 and 100) and thus some differing raw numbers show the same percent and non-0 raw numbers may show 0%

There was substantial variation in the preferred monitoring frequency for patients prescribed methotrexate, with a stepwise reduction in frequency over multiple stages generally preferred by most respondents (Table 3). Only 28 (13%) respondents reported using only one fixed monitoring schedule (most commonly every 3 months), while the remainder of respondents used two or more stages. For those using two stages (n = 88, 42%), most monitored more frequently in the first stage (most commonly monthly), followed by every three months thereafter. There was no relationship between ongoing monitoring rate and practitioner characteristics including sex, years of experience, practice setting, number of half-day sessions or number of estimated changes to csDMARD doses made per month.

**Table 3:** Frequency of laboratory monitoring of methotrexate in each stage of respondents’ preferred schedule by respondent percentage, N = 188^#^

Schedule (N, %)*	Weekly (N, %)*	Every 2 Weeks (N, %)*	Monthly (N, %)*	Every 2 Months (N, %)*	Every 3 Months (N, %)*	Every 4 Months (N, %)*	Every 6 Months (N, %)*	Yearly (N, %)*
1 Stage (27, 14%)								
Stage 1	0 (0%)	0 (0%)	7 (26%)	4 (15%)	13 (48%)	3 (11%)	0 (0%)	0 (0%)
2 Stages (82, 44%)								
Stage 1	1 (1%)	10 (12%)	63 (77%)	4 (5%)	4 (5%)	0 (0%)	0 (0%)	0 (0%)
Stage 2	0 (0%)	0 (0%)	7 (9%)	9 (11%)	61 (74%)	2 (2%)	1 (1%)	2 (2%)
3 Stages (63, 34%)								
Stage 1	4 (6%)	35 (56%)	23 (37%)	0 (0%)	0 (0%)	0 (0%)	1 (2%)	0 (0%)
Stage 2	0 (0%)	1 (2%)	33 (52%)	20 (32%)	8 (13%)	0 (0%)	1 (2%)	0 (0%)
Stage 3	0 (0%)	0 (0%)	0 (0%)	4 (6%)	51 (81%)	3 (5%)	3 (5%)	2 (3%)
4 Stages (16, 9%)								
Stage 1	0 (0%)	4 (25%)	11 (69%)	0 (0%)	1 (6%)	0 (0%)	0 (0%)	0 (0%)
Stage 2	0 (0%)	0 (0%)	6 (38%)	5 (31%)	4 (25%)	0 (0%)	1 (6%)	0 (0%)
Stage 3	0 (0%)	0 (0%)	3 (19%)	4 (25%)	6 (38%)	1 (6%)	2 (13%)	0 (0%)
Stage 4	0 (0%)	0 (0%)	2 (13%)	0 (0%)	10 (63%)	1 (6%)	2 (13%)	1 (6%)

^#^Frequency data unavailable for whole cohort (N = 221)

*Percentages are all rounded to the nearest whole number (including 0 and 100) and thus some differing raw numbers show the same percent and non-0 raw numbers may show 0%

Despite their varied side effect profiles, other csDMARDs were monitored in a similar pattern to methotrexate (Table 4A). Leflunomide monitoring was approximately the same as methotrexate for 90% of respondents. 75 (40%) respondents monitored sulfasalazine less frequently. Most participants reported that they did not change monitoring frequency when prescribing combination csDMARDs, except for the combination of methotrexate and leflunomide where most reported an increase in monitoring (Table 4B). An increase in the frequency of monitoring was reported for patients with comorbidities including fatty liver (69%) or other liver disease (73%), increased alcohol intake (60%), cytopenias (84%), or impaired renal function (70%, Table 4C). Age > 80 years old also led to an increase in monitoring in 45% of respondents.

**Table 4:** (A) Relative monitoring frequency for each csDMARD compared to methotrexate by respondent percentage, N = 187^#^; (B) change in monitoring frequency for each csDMARD combination by respondent percentage, N = 173^#^; (C) change in monitoring frequency for each co-morbidity by respondent percentage, N = 173^#^

A: csDMARD	More (N, %)*	Less (N, %)*	Equal (N, %)*	No monitoring (N, %)*	Not prescribed (N, %)*
(A)					
Leflunomide	8 (4%)	8 (4%)	168 (90%)	0 (0%)	3 (2%)
Sulfasalazine	12 (6%)	75 (40%)	97 (52%)	1 (1%)	2 (1%)
Hydroxychloroquine	0 (0%)	81 (43%)	20 (11%)	86 (46%)	0 (0%)
Azathioprine	33 (18%)	7 (4%)	141 (75%)	1 (1%)	5 (3%)
Mycophenolate	26 (14%)	11 (6%)	142 (76%)	0 (0%)	8 (4%)
Tacrolimus and other calcineurin inhibitors	30 (16%)	4 (2%)	81 (43%)	4 (2%)	68 (36%)

csDMARD combination	More (N, %)*	Less (N, %)*	Equal (N, %)*		Not prescribed (N, %)*
(B)					
Methotrexate/leflunomide	76 (44%)*	0 (0%)	73 (42%)		24 (14%)
Methotrexate/sulfasalazine	19 (11%)	2 (1%)	145 (84%)		7 (4%)
Methotrexate/hydroxychloroquine	4 (2%)	3 (2%)	166 (96%)		0 (0%)
Sulfasalazine/hydroxychloroquine	3 (2%)	16 (9%)	147 (85%)		7 (4%)
Leflunomide/hydroxychloroquine	6 (3%)	3 (2%)	154 (89%)		10 (6%)
Sulfasalazine/leflunomide	28 (16%)	2 (1%)	131 (76%)		12 (7%)

Co-morbidity	More (N, %)*	Less (N, %)*	Equal (N, %)*	Cease csDMARD (N, %)*	
(C)					
Obesity	26 (15%)*	1 (1%)	146 (84%)	0 (0%)	
Fatty liver disease	120 (69%)	2 (1%)	51 (29%)	0 (0%)	
Other liver disease	126 (73%)	2 (1%)	35 (20%)	10 (6%)	
Alcohol intake above NHMRC Guideline	104 (60%)	1 (1%)	47 (27%)	21 (12%)	
Existing cytopenias	145 (84%)	0 (0%)	19 (11%)	9 (5%)	
eGFR < 30	121 (70%)	1 (1%)	20 (12%)	31 (18%)	
eGFR 30–60	75 (43%)	3 (2%)	93 (54%)	2 (1%)	
18–60 years old	3 (2%)	8 (5%)	162 (94%)	0 (0%)	
60–80 years old	33 (19%)	4 (2%)	136 (79%)	0 (0%)	
> 80 years old	78 (45%)	2 (1%)	89 (51%)	4 (2%)	

^#^Change in monitoring frequency data unavailable for whole cohort (N = 221)

*Percentages are all rounded to the nearest whole number (including 0 and 100) and thus some differing raw numbers show the same percent and non-0 raw numbers may show 0%

Self-reported responses to changes in blood tests results over time were highly variable. When prescribing methotrexate, a majority (n = 94, 54%) of respondents reported that they would not act on trends in FBC or LFT if results were within the normal range, while 38 (22%) reported that they commonly would and 37 (21%) would sometimes. In a typical patient on a csDMARD (excluding hydroxychloroquine), moderate lymphopenia (0.2–0.5 10^9^/L) or a neutrophil count of 0.51–0.8 × 10^9^/L was the most common threshold for immediate suspension of a csDMARD (Additional file 2: Table S1). Respondents showed great variation in what abnormal liver transaminases would precipitate a reduction or suspension of csDMARD prescription (Table 5). The response to abnormal platelet and haemoglobin results was similar (Additional file 3: Table S2, Additional file 4: Table S3).

**Table 5:** Least severe event regarding ALT/AST monitoring precipitating a change in methotrexate prescription by respondent (%), N = 150^#^

Event	Change in prescription	Respondent (N, %)*
Any	None	0 (0%)
< 2ULN ALT/AST	Reduce dose	36 (24%)
< 2ULN ALT/AST	Suspend drug	9 (6%)
2–3 ULN ALT/AST	Reduce dose	29 (19%)
2–3 ULN ALT/AST	Suspend drug	16 (11%)
> 3 ULN ALT/AST	Reduce dose	6 (4%)
> 3 ULN ALT/AST	Suspend drug	12 (8%)
Multiple rising ALT/AST with most recent < 2 ULN ALT/AST	Reduce or suspend drug	19 (13%)
Multiple rising ALT/AST with most recent 2–3 ULN ALT/AST	Reduce or suspend drug	12 (8%)
Multiple rising ALT/AST with most recent > 3 ULN ALT/AST	Reduce or suspend drug	7 (5%)
Other	Individual	4 (3%)

^#^Impact of monitoring data unavailable for whole cohort (N = 221)

*Percentages are all rounded to the nearest whole number (including 0 and 100) and thus some differing raw numbers show the same percent and non-0 raw numbers may show 0%

Most considered that current guideline advice regarding the frequency of monitoring is about right (n = 83, 55%) but over a third (n = 56, 37%) considered that it was too frequent. Most respondents considered the frequency of monitoring for oral csDMARDs by rheumatologists to be about right (n = 87, 58%), while a minority considered it was either too frequent (n = 40, 27%) or not frequent enough (n = 8, 5%).

Male respondents were significantly more likely to feel that monitoring was being performed too frequently with “probably about right/too frequent/not frequent enough/other” responses of 48%/41%/6%/5%, and female respondents 65%/15%/5%/14% (*P* = 0.02). In logistic regression only female sex significantly affected this result (OR 3.4, 95%CI 1.5–7.6). This was also reflected in responses to the question about whether respondents felt the monitoring frequency recommended by guidelines was “probably about right/too frequent/not frequent enough” with males responding 64%/26%/1%, and females 44%/51%/2% (*P* < 0.01). In logistic regression only female sex significantly affected this result (OR 2.7, 95%CI 1.3–5.7).

Female respondents were significantly more likely to act on trends within normal ranges than male respondents, with “yes/no/not sure/sometimes” responses of 29%/45%/1%/25% for females and 15%/67%/2%17% for males (*P* < 0.01). Females also ceased csDMARDs for higher levels of lymphopenia compared to male respondents, 30%/44%/21%/5% (females) and 47%/42%/11%/0% (males) for < 0.02/ > 0.2– < 0.5/ > 0.5– < 0.8/ > 0.8 × 10^9^/L respectively (*P* = 0.02).

Most respondents reported ceasing or changing a csDMARD less than once per month (n = 63, 42%), followed by 1–2 changes per month (31%). In univariate linear regression there was no significant relationship between the number of outpatient consultation sessions per week worked and the number of times per month respondents would cease or change a csDMARD (adjusted r^2^ = 0.004, *P* = 0.21).

## Discussion

Our study has shown that rheumatologists and trainees are ordering consistent types of monitoring tests, but they are doing so in a wide variety of frequencies. Co-morbidities commonly increase monitoring frequency but only a combination of methotrexate and leflunomide generally increase monitoring frequency. There is also a wide variety of different actions, or inactions, in response to abnormal monitoring tests. Many feel that both the frequency of monitoring suggested by guidelines and the monitoring frequency being performed in clinical practice was too frequent.

Of interest, male respondents also reported greater tolerance of significant abnormalities in monitoring tests before acting. Compared with female respondents, male respondents also reported feeling that monitoring tests were being performed too frequently, and that recommended testing frequencies in guidelines were too frequent. This may reflect the sex differences in risk taking that have been observed when taking a range of different risks including hypothetical choices, financial risks, health risks and intellectual risk taking [13, 14].

Participants reported that they monitored some combinations of csDMARD more frequently, including methotrexate-leflunomide. Some studies, but not all, have shown that combining methotrexate and leflunomide causes an increased incidence of liver test abnormalities so the value of potentially increasing monitoring is unknown [15–18]. There was no relationship seen between the volume of clinical work a respondent did and the frequency that they changed csDMARDs dosing on their patients. This suggests that there exists substantial practice variation regarding dose changes of csDMARDs in response to abnormal monitoring tests.

The study was anonymous and therefore allowed respondents to not be impeded by any perceived scrutiny from others in expressing their opinions. The limitations of this work include the small size. Due to the design and distribution method of the study, the results may not be broadly representative, however the results paint a picture of wide variation even in this small sample. The study cannot examine practice only self-reported practice; although it seems very unlikely that wide variations in stated practice and attitudes would not result in wide variation in practice. Finally, while these results are the stated attitudes and behaviour of clinicians, they may not actually reflect, and likely do not accurately reflect, the monitoring being completed by patients. These limitations could be addressed by reminders to improve response rate, and performing studies where data is collected to assess the actual behaviour of clinicians and not just their opinions and attitudes.

Part of the impetus for this work included the daily experience of many rheumatologists who review monitoring tests which are normal or near normal and do not precipitate changes in csDMARD therapies. While it is important to monitor patients using csDMARDs for known adverse events such as methotrexate-induced liver fibrosis or neutropenia, it would seem evident that it is unclear what the optimal regimen is to both protect our patients but also make good use of limited health resources. With a prevalence of immune-mediated disease in the population of up to 9%, the use of immune modulating drugs is widespread, estimated at around 2.8% of the US insured population [19]. Laboratory monitoring consumes not only direct financial costs, but the time of patients and clinicians to order, collect and review the tests [20]. This work has potential relevance for other medical specialities that have extensive use of immune-modulating drugs such as immunology, gastroenterology, dermatology, and neurology.

Recent work from a United Kingdom primary care study found that the cumulative incidence of methotrexate discontinuation is 1 in 24 in the first year for abnormal tests and 1 in 169 for severely abnormal tests [21]. This rate decreases to 1 in 45 and 1 in 352 for abnormal and severely abnormal tests respectively per year thereafter. Equivalent leflunomide discontinuation rates are lower. This would suggest that severely abnormal blood tests precipitating discontinuation are uncommon. There is a definite ongoing need for monitoring tests, but the value of current monitoring regimens is currently uncertain.

Efforts to improve the value of care that is provided in medicine have been widespread [22]. In rheumatology this has included the Choosing Wisely projects in Australian, US and Canadian rheumatology [23–25]. If we are to afford new, and often more expensive technology, for the benefit of our patients, we have to ensure we are providing the best value for money with our current resources [10].

The most important next step is to determine through high quality clinical trials the optimal monitoring frequency for each drug, co-morbidity and combination. This work can then support high quality evidence-based guidelines that has the potential to lead to improvements in the quality and value of care we provide to our patients.

## Supplementary Information

**Additional file 1.** csDMARD Monitoring Survey.

**Additional file 2. Supplementary Table 1**. Least severe event regarding neutrophils or lymphocytes causing a corresponding change in methotrexate prescription by respondent percentage, N = 150.

**Additional file 3. Supplementary Table 2**. Least severe event regarding thrombocytopenia precipitating an immediate suspension of the prescribed csDMARD, N = 150.

**Additional file 4. Supplementary Table 3**. Least severe event regarding anaemia precipitating an immediate suspension of the prescribed csDMARD, N = 150.

## Data Availability

The datasets used and/or analysed during the current study available from the corresponding author on reasonable request.
