# Supplementary material for: Attitudes and practices in the laboratory monitoring of conventional synthetic disease modifying anti-rheumatic drugs by rheumatologists and rheumatology trainees
Source: BMC Rheumatol. 2022 Oct 17;6:59. doi: 10.1186/s41927-022-00290-y (PMC9575262; doi:10.1186/s41927-022-00290-y)
Supplement: Supplementary file 1 — Additional file 1. csDMARD Monitoring Survey. [file 41927_2022_290_MOESM1_ESM.pdf]

csDMARD Monitoring Survey

Please consider completing this short survey on lab monitoring of rheumatology DMARDs, it is the short and just asks about your current practice in relation to monitoring common DMARDs. The survey is anonymous and is being completed by A/Prof Philip Robinson from the Royal Brisbane Hospital. If you proceed to complete the survey we take this as consent to participate. This survey has been approved by the Royal Brisbane & Women's Hospital Human Research Ethics Committee.

csDMARD Monitoring Survey

* 1. In a typical patient, do you perform thiopurine methyltransferase (TPMT) genotype testing prior to commencing azathioprine?

- ◯ Yes
- ◯ No
- ◯ Sometimes
- ◯ I never prescribe azathioprine

* 2. In an uncomplicated patient, what is your approach to screening for hydroxychloroquine retinal toxicity?

- ◯ No regular screening
- ◯ Yearly eye checks from time of initiation
- ◯ Baseline, then yearly screening after 5 years in those with no risk factors
- ◯ Follow advice of ophthalmologist or optometrist
- ◯ I don't prescribe hydroxychloroquine
- ◯ Other (please specify)

[ ]

* 3. In an uncomplicated patient, what regular monitoring tests do you order or recommend to be ordered for each drug specified below?

	Complete Blood Count	Creatinine/eGFR	Liver Function Tests	I don't monitor blood tests with this drug	I don't prescribe this drug
Methotrexate	☐	☐	☐	☐	☐
Leflunomide	☐	☐	☐	☐	☐
Sulfasalazine	☐	☐	☐	☐	☐
Hydroxychloroquine	☐	☐	☐	☐	☐
Azathioprine	☐	☐	☐	☐	☐
Mycophenolate	☐	☐	☐	☐	☐
Tacrolimus and other calcineurin inhibitors	☐	☐	☐	☐	☐

* 4. When prescribing methotrexate to an uncomplicated patient, how many different periods of monitoring are there (e.g. every week for 1 month, then every month thereafter is 2 periods; while every week regardless is 1 period)?

	Number of Periods
Answers	[ ]

# csDMARD Monitoring Survey

* 5. In an uncomplicated patient, what is the frequency of fixed monitoring for methotrexate?

* 6. In an uncomplicated patient, what is the length and frequency of monitoring for each period when prescribing methotrexate?

	Length of period	Monitoring Frequency
Period 1		
Period 2		

* 7. In an uncomplicated patient, what is the length and frequency of monitoring for each period when prescribing methotrexate?

	Length of period	Monitoring Frequency
Period 1		
Period 2		
Period 3		

* 8. In an uncomplicated patient, what is the length and frequency of monitoring for each period when prescribing methotrexate?

	Length of period	Monitoring Frequency
Period 1		
Period 2		
Period 3		
Period 4		

* 9. In an uncomplicated patient, what is the relative frequency of monitoring (compared to methotrexate) for each of the following drugs?

	Relative frequency
Leflunomide	
Sulfasalazine	
Hydroxychloroquine	
Azathioprine	
Mycophenolate	
Tacrolimus and other calcineurin inhibitors	

csDMARD Monitoring Survey

* 10. In an uncomplicated patient, what effect on monitoring frequency does the following combinations have in most patients?

	Increased Frequency	Decreased Frequency	No Change	I don't use the combination
MTX/Leflunomide	○	○	○	○
MTX/SSZ	○	○	○	○
MTX/HCQ	○	○	○	○
SSZ/HCQ	○	○	○	○
Leflunomide/HCQ	○	○	○	○
SSZ/Leflunomide	○	○	○	○

* 11. In an otherwise uncomplicated patient, what effect on monitoring frequency does the following co-morbidities have?

	Increased frequency	Decreased frequency	No change	I would not use DMARDs
Obesity	○	○	○	○
Fatty liver disease	○	○	○	○
Other liver disease	○	○	○	○
Alcohol intake above NHMRC guidelines	○	○	○	○
Existing cytopenias	○	○	○	○
eGFR < 30	○	○	○	○
eGFR 30-60	○	○	○	○
18 - 60 years old	○	○	○	○
60 - 80 years old	○	○	○	○
>80 years old	○	○	○	○

* 12. When prescribing Methotrexate to an uncomplicated patient, do you act on trends in the results of your full blood count and liver test monitoring even if within normal ranges?

- ○ Yes
- ○ No
- ○ Not sure
- ○ Sometimes

* 13. Do you delegate responsibility for monitoring to the GP in a “shared care” arrangement?

- Always
- Frequently
- Occasionally
- Never

* 14. If you do delegate this monitoring responsibility, do you find that the monitoring is generally carried out as recommended by you to the delegate?

- Always
- Frequently
- Occasionally
- Never

* 15. In an uncomplicated patient; considering csDMARDS but excluding hydroxychloroquine, what is the highest level of lymphopenia that would generally precipitate you to suspend a medication immediately?

- <0.2
- >0.2 - <0.5
- >0.5 - <0.8
- >0.8

* 16. In an uncomplicated patient; considering csDMARDS but excluding hydroxychloroquine, what is the highest level of neutropenia that would generally precipitate you to suspend a medication immediately?

- <0.2
- >0.2 - <0.5
- >0.2 - <0.8
- >0.8

* 17. In an uncomplicated patient; considering csDMARDS but excluding hydroxychloroquine, what is the highest level of thrombocytopenia that would generally precipitate you to suspend a medication immediately?

- <50
- >50 - <100
- >100 - <200
- >200 - <300
- >300 - <450
- Other (please specify)

* 18. In an uncomplicated patient; considering csDMARDS but excluding hydroxychloroquine, what is the highest level of anaemia that would generally precipitate you to suspend a medication immediately?

- <50 g/L
- >50 to < 75 g/L
- >75 to <100 g/L
- >100 g/L to Lower limit of normal for the patient

* 19. Do you think the frequency of blood test monitoring for oral csDMARDs as currently practiced by most rheumatologists is:

- Probably about right
- Too frequent
- Not frequent enough
- I'm unfamiliar with the typical monitoring of most rheumatologists

* 20. Do you think the frequency of blood test monitoring for oral csDMARDs as currently recommended by most guidelines is:

- Probably about right
- Too frequent
- Not frequent enough
- I'm unfamiliar with the current recommendations for csDMARD monitoring in most guidelines

* 21. In an uncomplicated patient on MTX for rheumatoid arthritis causing an abnormal ALT thought to be likely due to the MTX, what most accurately describes what your behaviour would be? Choose the least severe option that precipitates you to act

- Don't suspend the drug ever
- Reduce dose for new abnormalities (> ULN -<2 ULN ALT/AST)
- Suspend drug for new abnormalities (>ULN - < 2ULN ALT/AST)
- Reduce dose for new abnormalities (>2 - <3 ULN ALT/AST)
- Suspend drug for new abnormalities (>2 - <3 ULN ALT/AST)
- Reduce dose for new abnormalities (>3 ULN ALT/AST)
- Suspend drug for new abnormalities (>3 ULN ALT/AST)
- Only reduce dose or suspend drug if a trend of rising ALT/AST levels over multiple tests (most recent test is >ULN - < 2 ULN ALT/AST)
- Only reduce dose or suspend drug if a trend of rising ALT/AST levels over multiple tests (most recent test is >2- <3 ULN ALT/AST)
- Only reduce dose or suspend drug if a trend of rising ALT/AST levels over multiple tests (most recent test is >3 ULN ALT/AST)
- Other (please specify)

* 22. In an uncomplicated patient on MTX for rheumatoid arthritis causing a low neutrophils or lymphocytes thought to be likely due to csDMARDs, what most accurately describes what your behaviour would be? Choose the least severe option that precipitates you to act

- Don't suspend the drug ever
- Reduce dose for new abnormalities (Neutrophils or Lymphocytes <0.4)
- Suspend drug for new abnormalities (Neutrophils or Lymphocytes <0.4)
- Reduce dose for new abnormalities (Neutrophils or Lymphocytes >0.4 but <0.6)
- Suspend drug for new abnormalities (Neutrophils or Lymphocytes >0.4 but <0.6)
- Reduce dose for new abnormalities (Neutrophils or Lymphocytes >0.6 but <0.8)
- Suspend drug for new abnormalities (Neutrophils or Lymphocytes >0.6 but <0.8)
- Reduce dose for new abnormalities (Neutrophils or Lymphocytes >0.8 but <1.0)
- Suspend drug for new abnormalities (Neutrophils or Lymphocytes >0.8 but <1.0)
- Only reduce dose or suspend drug if a trend of falling Neutrophils or Lymphocytes levels over multiple tests (most recent test is Neutrophils or Lymphocytes <0.4)
- Only reduce dose or suspend drug if a trend of falling Neutrophils or Lymphocytes levels over multiple tests (most recent test is Neutrophils or Lymphocytes >0.4 but <0.6)
- Only reduce dose or suspend drug if a trend of falling Neutrophils or Lymphocytes levels over multiple tests (most recent test is Neutrophils or Lymphocytes >0.6 but <0.8)
- Only reduce dose or suspend drug if a trend of falling Neutrophils or Lymphocytes levels over multiple tests (most recent test is Neutrophils or Lymphocytes >0.8 but <1.0)

* 23. How many times a month do you cease or change dose of a medication due to csDMARD monitoring bloods?

- <1 time per month
- 1-2 times per month
- 3-5 times per month
- 6-10 times per month
- >10 times per month

csDMARD Monitoring Survey

* 24. In what country do you work?

* 25. Are you female, male or other?

- Female
- Male
- Other

* 26. How many years in rheumatology practice have you had as an advanced trainee or higher?

- <2
- 2-5
- 6-10
- 11-20
- >20

* 27. In which setting do you perform your clinical work?

- Mostly private practice
- Mostly public hospital practice
- An even mix of private and public

* 28. How many half day outpatient consulting sessions where you care for patients on average do you complete per week?
