# Supplementary material for: Attitudes and practices in the laboratory monitoring of conventional synthetic disease modifying anti-rheumatic drugs by rheumatologists and rheumatology trainees
Source: BMC Rheumatol. 2022 Oct 17;6:59. doi: 10.1186/s41927-022-00290-y (PMC9575262; doi:10.1186/s41927-022-00290-y)
Supplement: Supplementary file 2 — Additional file 2. Supplementary Table 1. Least severe event regarding neutrophils or lymphocytes causing a corresponding change in methotrexate prescription by respondent percentage, N = 150. [file 41927_2022_290_MOESM2_ESM.docx]

**Supplementary Table 1:** Least severe event regarding neutrophils or lymphocytes causing a corresponding change in methotrexate prescription by respondent percentage, N = 150^#^

| Event | Change in Prescription | Respondent (N, %)* |
| --- | --- | --- |
| Any | None | 3 (2%) |
| Neutrophils or Lymphocytes <0.4 | Reduce Dose | 21 (14%) |
| Neutrophils or Lymphocytes <0.4 | Suspend Drug | 19 (13%) |
| Neutrophils or Lymphocytes 0.4-0.6 | Reduce Dose | 17 (11%) |
| Neutrophils or Lymphocytes 0.4-0.6 | Suspend Drug | 10 (7%) |
| Neutrophils or Lymphocytes 0.61-0.8 | Reduce Dose | 13 (9%) |
| Neutrophils or Lymphocytes 0.61-0.8 | Suspend Drug | 11 (7%) |
| Neutrophils or Lymphocytes 0.81-1 | Reduce Dose | 12 (8%) |
| Neutrophils or Lymphocytes 0.81-1 | Suspend Drug | 14 (9%) |
| Multiple Falling Neutrophil/ Lymphocyte with most recent <0.4 | Reduce or Suspend Drug | 10 (7%) |
| Multiple Falling Neutrophil/ Lymphocyte with most recent 0.4-0.6 | Reduce or Suspend Drug | 1 (1%) |
| Multiple Falling Neutrophil/ Lymphocyte with most recent 0.61-0.8 | Reduce or Suspend Drug | 4 (3%) |
| Multiple Falling Neutrophil/ Lymphocyte with most recent 0.81-1 | Reduce or Suspend Drug | 15 (10%) |

# Neutrophil and lymphocyte monitoring data unavailable for whole cohort (N=221)

* Percentages are all rounded to the nearest whole number (including 0 and 100) and thus some differing raw numbers show the same percent and non-0 raw numbers may show 0%.
