# Supplementary material for: Attitudes and practices in the laboratory monitoring of conventional synthetic disease modifying anti-rheumatic drugs by rheumatologists and rheumatology trainees
Source: BMC Rheumatol. 2022 Oct 17;6:59. doi: 10.1186/s41927-022-00290-y (PMC9575262; doi:10.1186/s41927-022-00290-y)
Supplement: Supplementary file 3 — Additional file 3. Supplementary Table 2. Least severe event regarding thrombocytopenia precipitating an immediate suspension of the prescribed csDMARD, N = 150. [file 41927_2022_290_MOESM3_ESM.docx]

**Supplementary** **Table 2:** Least severe event regarding thrombocytopenia precipitating an immediate suspension of the prescribed csDMARD, N = 150^#^

| Thrombocytopenia threshold (10^3^/µL) | Respondent (N, %)* |
| --- | --- |
| ≤50 | 42 (28%) |
| >50 to ≤100 | 88 (59%) |
| >100 to ≤200 | 18 (12%) |
| >200 to ≤300 | 0 (0%) |
| >300 | 1 (1%) |
| Other | 1 (1%) |

# Laboratory monitoring data unavailable for whole cohort (N=221)

* Percentages are all rounded to the nearest whole number (including 0 and 100) and thus some differing raw numbers show the same percent and non-0 raw numbers may show 0%.
