# Supplementary material for: Attitudes and practices in the laboratory monitoring of conventional synthetic disease modifying anti-rheumatic drugs by rheumatologists and rheumatology trainees
Source: BMC Rheumatol. 2022 Oct 17;6:59. doi: 10.1186/s41927-022-00290-y (PMC9575262; doi:10.1186/s41927-022-00290-y)
Supplement: Supplementary file 4 — Additional file 4. Supplementary Table 3. Least severe event regarding anaemia precipitating an immediate suspension of the prescribed csDMARD, N = 150. [file 41927_2022_290_MOESM4_ESM.docx]

**Supplementary** **Table 3:** Least severe event regarding anaemia precipitating an immediate suspension of the prescribed csDMARD, N = 150^#^

| Heamoglobin level threshold (g/L) | Respondent (N, %)* |
| --- | --- |
| ≤25 | 0 (0%) |
| >25 to ≤50 | 12 (8%) |
| >50 to ≤75 | 62 (41%) |
| >75 to ≤100 | 69 (46%) |
| >100 to lower limit of normal for the patient | 7 (5%) |

# Laboratory monitoring data unavailable for whole cohort (N=221)

* Percentages are all rounded to the nearest whole number (including 0 and 100) and thus some differing raw numbers show the same percent and non-0 raw numbers may show 0%.
